# Gene Expression and Methylation Analyses Suggest *DCTD* as a Prognostic Factor in Malignant Glioma

**DOI:** 10.1038/s41598-017-11962-y

**Published:** 2017-09-14

**Authors:** Huimin Hu, Zheng Wang, Mingyang Li, Fan Zeng, Kuanyu Wang, Ruoyu Huang, Haoyuan Wang, Fan Yang, Tingyu Liang, Hua Huang, Tao Jiang

**Affiliations:** 1 Department of Molecular Neuropathology, Beijing Neurosurgical Institute, Capital Medical University, Beijing, China; 2 Chinese Glioma Cooperative Group (CGCG), Beijing, China; 3 Department of Neurosurgery, Beijing Tiantan Hospital, Capital Medical University, Beijing, China; 4 Center of Brain Tumor, Beijing Institute for Brain Disorders, Beijing, China; 5 Southern Medical University, Guangzhou, China

## Abstract

Malignant glioma is the most common brain cancer with dismal outcomes. Individual variation of the patients’ survival times is remarkable. Here, we investigated the transcriptome and promoter methylation differences between patients of malignant glioma with short (less than one year) and the patients with long (more than three years) survival in CGGA (Chinese Glioma Genome Atlas), and validated the differences in TCGA (The Cancer Genome Atlas) to identify the genes whose expression levels showed high concordance with prognosis of glioma patients, as well as played an important role in malignant progression. The gene coding a key enzyme in genetic material synthesis, *dCMP deaminase* (*DCTD*), was found to be significantly correlated with overall survival and high level of *DCTD* mRNA indicated shorter survival of the patients with malignant glioma in different databases. Our finding revealed *DCTD* as an efficient prognostic factor for malignant glioma. As DCTD inhibitor gemcitabine has been proposed as an adjuvant therapy for malignant glioma, our finding also suggests a therapeutic value of gemcitabine for the patients with high expression level of *DCTD*.

## Introduction

Glioma is the most common primary intracranial tumor, accounting for 46% of all intracranial tumors, and 2% of all adult cancers^1^. The WHO classification of central nervous system tumors (2007, fourth edition) divided diffuse glioma into WHO II, III and IV grades^2^. High-grade diffuse gliomas (WHO grade III and IV) and low-grade diffuse gliomas (WHO grade II) vary widely in tumor pathological morphology (such as collagen fiber content and morphological diversity), tumor development and prognosis of patients. Patients with glioblastoma (GBM, WHO grade IV), the most invasive glioma^3^, have the poorest prognosis, with a median overall survival of only 12–14 months, and a 5-year survival rate of only 9%^4^. The five-year survival rate for WHO grade III gliomas is 30%. The low-grade gliomas (WHO II grade) have a five-year survival rate of as high as 50%^5^. Clinically, WHO III and IV grade gliomas, which were characterized by strong invasion and significantly short survival are collectively mentioned as malignant glioma^6^.

The current standard treatment for malignant glioma is surgical resection followed by radiotherapy combined with concurrent and/or adjuvant temozolomide (TMZ) chemotherapy^7–9^. However, a large number of clinical studies have indicated that only approximately 9% of malignant glioma patients who received standard treatment could survive more than 5 years^4^. Although many studies have been conducted to improve the treatment of malignant glioma and to facilitate the increase of patients’ survival time, there have been no newly found effective treatments. Since the year of 2015, immunotherapeutic approaches have made remarkable progresses in hematopoietic tumors. However, the immunotherapeutic clinical trials in solid tumors including glioma are far from satisfying^10^. Antibody-drugs are currently explored to cure glioma while quite a number of challenges still exists^11^. Sustaining lag in the therapeutic approaches development is due to the limited understanding of the extremely complex networks of genomic alterations and molecular regulations controlling the initiation and development of malignant gliomas.

It is noteworthy that although the prognosis of malignant glioma patients remains generally poor, individual variation of the patients’ survival times is remarkable. Significantly, different outcomes reflect the intrinsic different expression level of the critical oncogenic genes or genomic alteration. Exploration of the intrinsic differences between malignant glioma with long survival and those with short survival may help us to reveal efficient predictive factors of survival time and potential therapeutic targets.

To explore efficient prognostic factor and effective therapeutic targets, we analyzed mRNA expression and methylation datasets to screen overall survival (OS)-correlated genes by shuttling between datasets from TCGA (The Cancer Genome Atlas) and CGGA (Chinese Glioma Genome Atlas). Seven genes including *DCTD* (dCMP deaminase) passed the filtering criteria. The prognostic efficiency of *DCTD* expression level was validated in another two databases and the oncogenic features of *DCTD* were revealed and verified in four independent databases.

DCTD is a key enzyme in genetic material synthesis and taking charge of conversion of deoxycytidylate (dCMP) to deoxy-uridine monophosphate (dUMP). dUMP is a synthetic substrate of thymidylate^12^. Abnormal expression of *DCTD* would affect the stability of genetic material synthesis, which is vital important for rapid tumor expansion. We speculate that DCTD acts as a “biosynthetic catalyst” in cancer progression to meet the rapid cell proliferation and active demand for genetic material. This also suggests that the oxycytidine analog antimetabolite gemcitabine, an inhibitor of DCTD, might be an efficient drug for the treatment of patients with high level of *DCTD* transcription.

## Results

### Screening for critical genes in glioma origin or development through gene expression and methylation analyses

**Figure 1:**
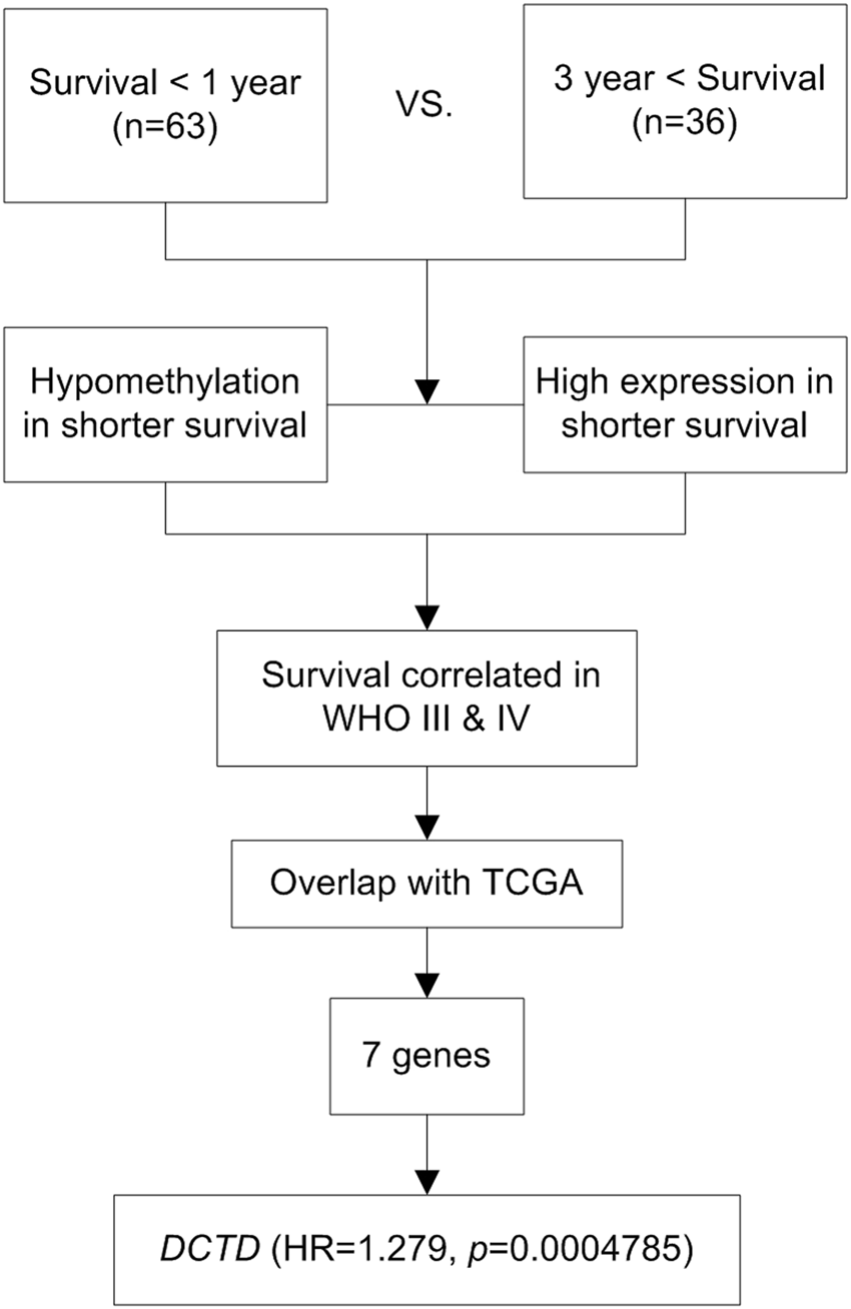
Data analysis pipeline to search for the OS-correlated critically important genes. The differences in the transcriptome between the WHO grade III and IV patients in CGGA database who lived for less than 1 year (n = 63) and those who lived for more than 3 years (n = 36) after diagnosis were analyzed. The level of gene promoter methylation in these patients was also compared. Gene lists derived based on transcriptional level and promoter methylation level was overlapped. The efficacy of the overlapping genes in predication of prognosis of all of the patients with WHO grade III and IV (except for the screening group, additional patients whose survival time is between 1 to 3 years were included, and the total number of samples was 178) glioma were tested. The genes with capability of predicting the survival length of malignant glioma patients were reserved. The prognosis effects of these genes in TCGA microarray data for GBM (n = 476) were tested. Only 7 genes, including *DCTD* were significantly correlated with the survival length of the TCGA GBM patients.

To search for the critical genes in glioma origin or development, we firstly investigated the global transcriptome differences (target proportion of false discoveries: 0.1, number of permutations: 100, percentile for determining called genes that are false: 90) between patients with WHO grade III and IV glioma surviving for less than 1 year (n = 63) and those surviving for more than 3 years (n = 36) after diagnosis using data from the Chinese Glioma Genome Atlas (CGGA) database (Table 1). Subsequently, the gene promoter methylation levels of these patients were also compared. Therefore, we derived a cluster of genes with hypomethylated promoter and a high level of transcription correlated with short survival time (Cox proportional hazards model, nominal significance level of each univariate test: 0.001). The efficacy of these genes in distinguishing prognosis of all the patients with WHO grade III and IV glioma (except for the screening group, additional patients whose survival time is between 1 to 3 years were included, and the total number of samples was 178) were tested (Cox proportional hazards model, nominal significance level of each univariate test: 0.001). So far, we had identified genes with promoter methylation and transcriptional levels related to patients’ OS and were able to predict the survival time of the malignant glioma patients. Next, we validated the correlation between prognosis and the expression levels of these genes in TCGA microarray database for GBM (n = 476, Cox proportional hazards model, nominal significance level of each univariate test: 0.001). Only seven genes were found to be significantly correlated with the survival of the TCGA GBM patients (Fig. 1). *DCTD* (*dCMP deaminase*) encoding critical enzyme in genetic material synthesis was among the 7 filtered genes (Table 2). The hazard ratio for survival of *DCTD* expression in TCGA microarray (n = 476) was 1.279 and the parametric p-value was 0.0004785 (significance of correlation between expression level and OS generated by BRB Array Tools) (Fig. 1).

**Table 1:** Clinical information of the patients in gene expression and methylation analyses.

	Survival <1 year	Survival >3 years
Age (year)	50.0* (13–70)	41.5* (17–66)
Gender		
Male	40	23
Female	23	13
OS (day)	231.0* (27–363)	1596.5* (1121–2257)
Grade		
WHOIII	11	21
WHOIV	52	15
Histology		
AA	6	3
AO	1	9
AOA	4	9
GBM	52	15

^*^Median value.

**Table 2:** The 7 filtered genes through gene expression and methylation Analyses.

Symbol	Parametric p-value	Hazard Ratio	FDR
EFEMP2	8e-07	1.272	0.000108
FBXO17	4.67e-05	1.279	0.0026
PDPN	5.78e-05	1.133	0.0026
BICD1	0.0001173	1.461	0.00396
DCTD	0.0004785	1.279	0.0129
PTRF	0.0007646	1.203	0.0172
MEOX2	0.0009865	1.085	0.019

### mRNA-level of *DCTD* could predict OS of the patients with malignant glioma

**Figure 2:**
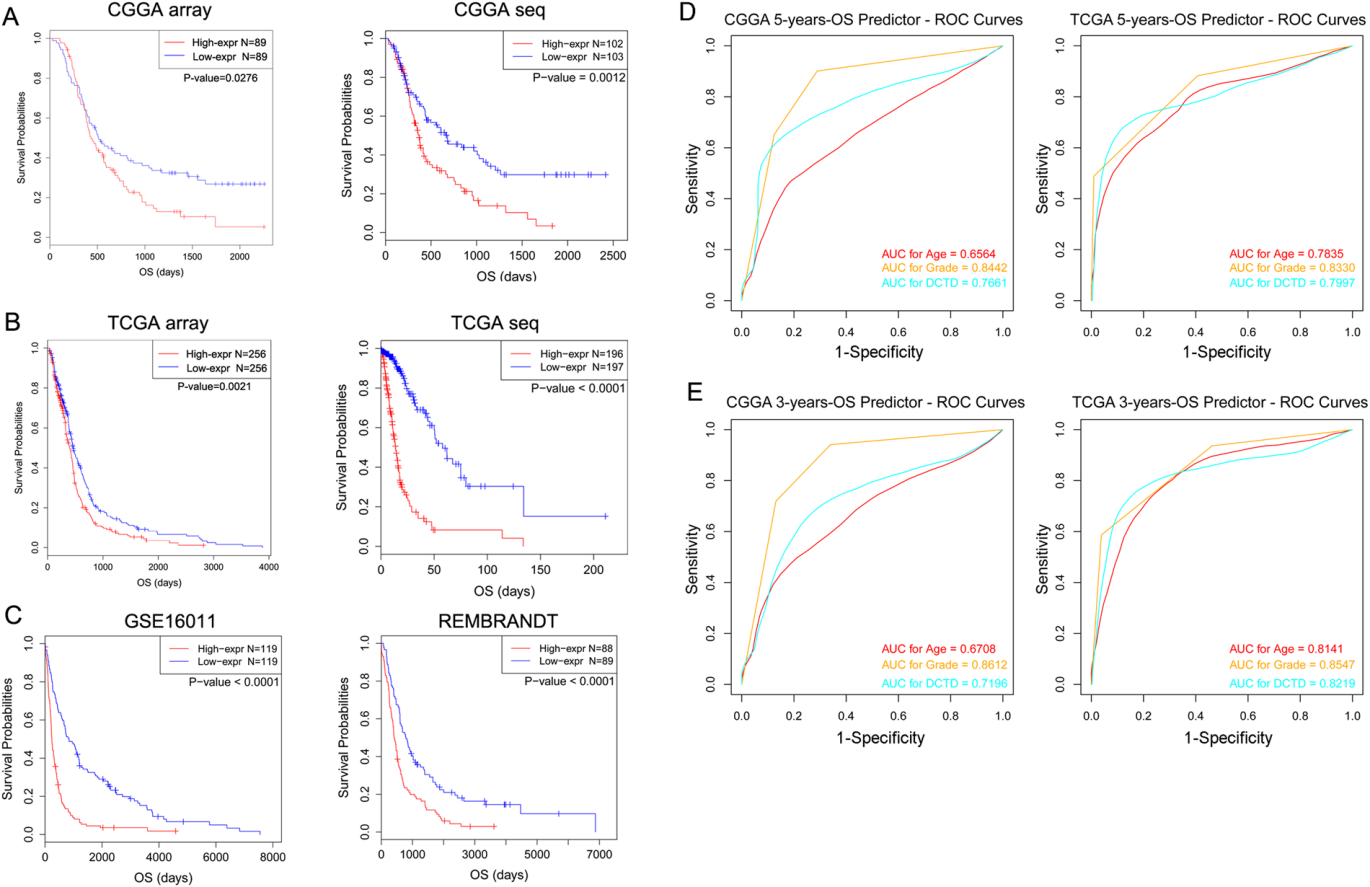
The prognosis efficiency of *DCTD*. (**A**) The prognosis efficiency of *DCTD* in all WHO grade III and IV (except for the screening group, additional patients whose survival time is between 1 to 3 years were included, and the total number of samples was 178) patients in CGGA transcriptional microarray data and WHO grade III and IV glioma in CGGA RNA-seq data. (**B**) The prognosis efficiency of *DCTD* in GBM (n = 512) from TCGA transcriptional microarray data that were updated in 2013, which was expanded from the dataset that we used to search for the 7 genes and in GBM (n = 393) from TCGA RNA-seq dataset. (**C**) The prognosis efficiency of *DCTD* validated in GSE16011 and REMBRANDT datasets. (**D**) The ROC curves indicating the sensitivity and specificity of predicting 5 years of survival with *DCTD*-level in CGGA and TCGA database. (**E**) The ROC curves indicating the sensitivity and specificity of predicting 3 years of survival with *DCTD*-level in CGGA and TCGA database.

**Figure 3:**
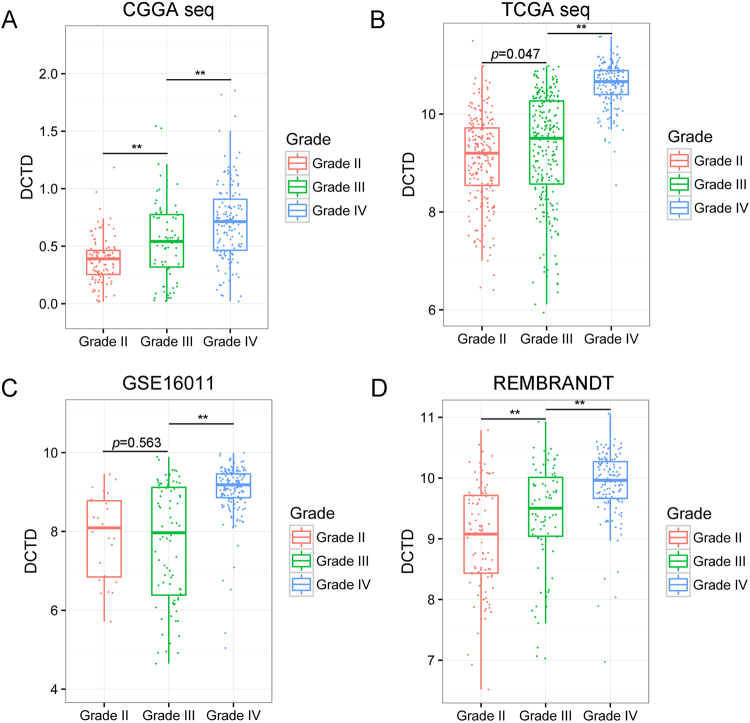
The correlation of *DCTD* expression level and WHO grade. *DCTD* expression levels in glioma of WHO grade II-IV in CGGA RNA-seq (**A**), TCGA RNA-seq (**B**), GSE16011 (**C**) and REMBRANDT (**D**) databases. **p < 0.01.

*DCTD* expression level was sufficient and efficient to predict the survival time of patients with malignant glioma. We tested the efficiency of *DCTD* expression level in prognosis of all patients with WHO grade III and IV glioma (except for the screening group, additional patients whose survival time is between 1 to 3 years were included, and the total number of samples was 178) in CGGA mRNA-array data. Half of the patients with relatively higher *DCTD* expression had marked poor outcomes. The predicative efficiency is also sound in CGGA mRNA-seq data (Fig. 2A). We next validated the efficiency of *DCTD* as a prognosis indicator in TCGA GBM transcriptional microarray data (n = 512) updated in 2013, which were expanded from the dataset that we used to screen for the above-mentioned 7 genes (Fig. 1). As expected, the efficiency of the *DCTD* transcriptional level as a prognosis indicator is also ideal (Fig. 2B). The OS indicator role of *DCTD* was validated in TCGA mRNA-seq (Fig. 2B), GSE16011 and REMBRANDT (Fig. 2C) data. The specificity and sensitivity of *DCTD* mRNA-level in predication of 5 (Fig. 2D) or 3 (Fig. 2E) years of survival was tested in CGGA and TCGA mRNA-seq data, and compared with “age” and “grade”. The area under curve (AUC) for *DCTD* transcriptional level in prediction of 5 and 3 years of survival in CGGA are 0.7661 and 0.7196 respectively. Those AUCs in TCGA are 0.7997 and 0.8219. The AUCs for *DCTD* mRNA-level are all larger than those of “age” in all of the four ROC tests, despite smaller than the AUCs of “grade”.

### *DCTD* expression level is correlated with glioma grade and shows a subtype preference

The intensive expression of *DCTD* in glioma patients with poorer survival suggests oncogenic features of this gene. To further understand whether *DCTD* plays a role in malignant progression of glioma, we compared the expression levels of *DCTD* in different WHO grades glioma derived from four datasets. Except for the differentiation between grade II and III in GSE16011 (*p = *0.563), *DCTD* expression increased along with grade progression, and the differentiations are all significant (*p < *0.05) (Fig. 3). This suggests that *DCTD* might play a part in malignant progression of low grade glioma.

**Figure 4:**
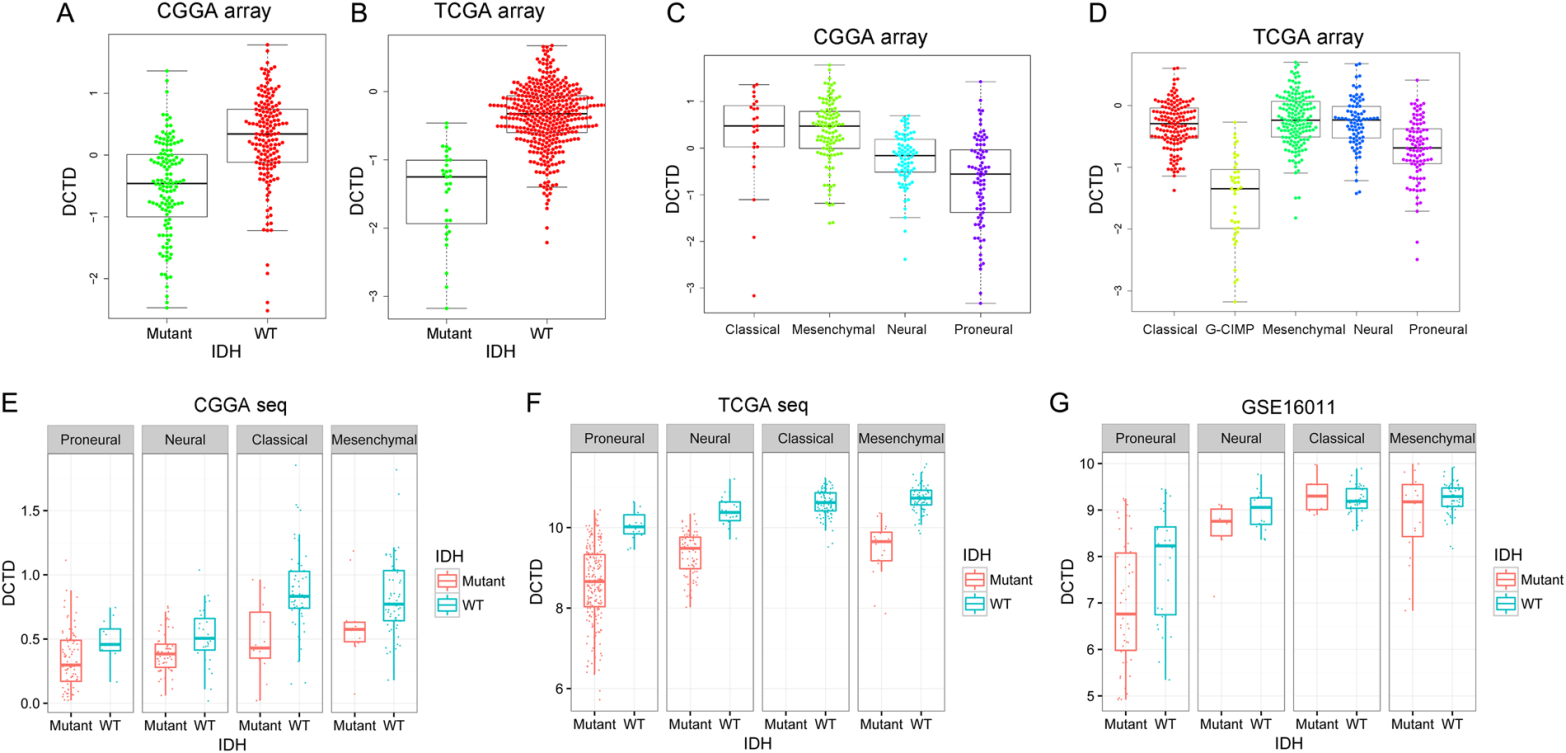
Correlation between *DCTD* expression level and *IDH1* mutation and the subtype preference. (**A**,**B**) Correlation of *DCTD* transcription level and *IDH1* mutation in CGGA (**A**) and TCGA (**B**) RNA microarray data. (**C**,**D**) Correlation of *DCTD* transcription level and transcriptomic subtype classification in CGGA (**C**) and TCGA (**D**) RNA microarray data. (**E**–**G**) Correlation of *DCTD* transcription level and *IDH1* mutation in different subtypes of glioma in CGGA RNA-seq (**E**), TCGA RNA-seq (**F**, no sample of classical subtype in TCGA seq data harbors IDH1 mutation) data and GSE16011 (**G**).

As *IDH1* mutation is a critical driver and prognosis indicator of glioma^4, 13^, we further explored the correlation between *DCTD* transcription level and *IDH1* mutation. Both in CGGA (all grades, n = 302) and TCGA (GBM, n = 543) datasets, the patients harboring *IDH1* mutation showed much lower expression of *DCTD* than those with wild-type *IDH1* (Fig. 4A,B). The correlation between *DCTD* expression level and glioma subtype could also reflect the prognosis efficacy of *DCTD*. *DCTD* expression levels in the four transcriptional characteristic subtypes were quite different in the CGGA dataset (all grades, n = 302, Fig. 4C). Patients of classical subtype or mesenchymal subtype primarily had strong *DCTD* expression. In TCGA (GBM, n = 543) data, patients with higher *DCTD* expression were concentrated in classical, mesenchymal and neural subtypes, whereas patients with lower *DCTD* expressions primarily belonged to G-CIMP or proneural subtypes (Fig. 4D), which are typically associated with better outcomes^14^. As validation, the correlations between *DCTD* transcriptional level and *IDH1* mutation or subtype were analyzed in RNA sequencing data derived from CGGA (Fig. 4E) or TCGA (Fig. 4F), as well as in GSE16011 (Fig. 4G). Except for the classical subtype of GSE16011 (the median of *DCTD* expression in *IDH1*-mutated glioma is higher than the ones with wild-type *IDH1*), all the analysis results were corresponding to above conclusions.

### *DCTD*-related genomic alterations and biological processes

**Figure 5:**
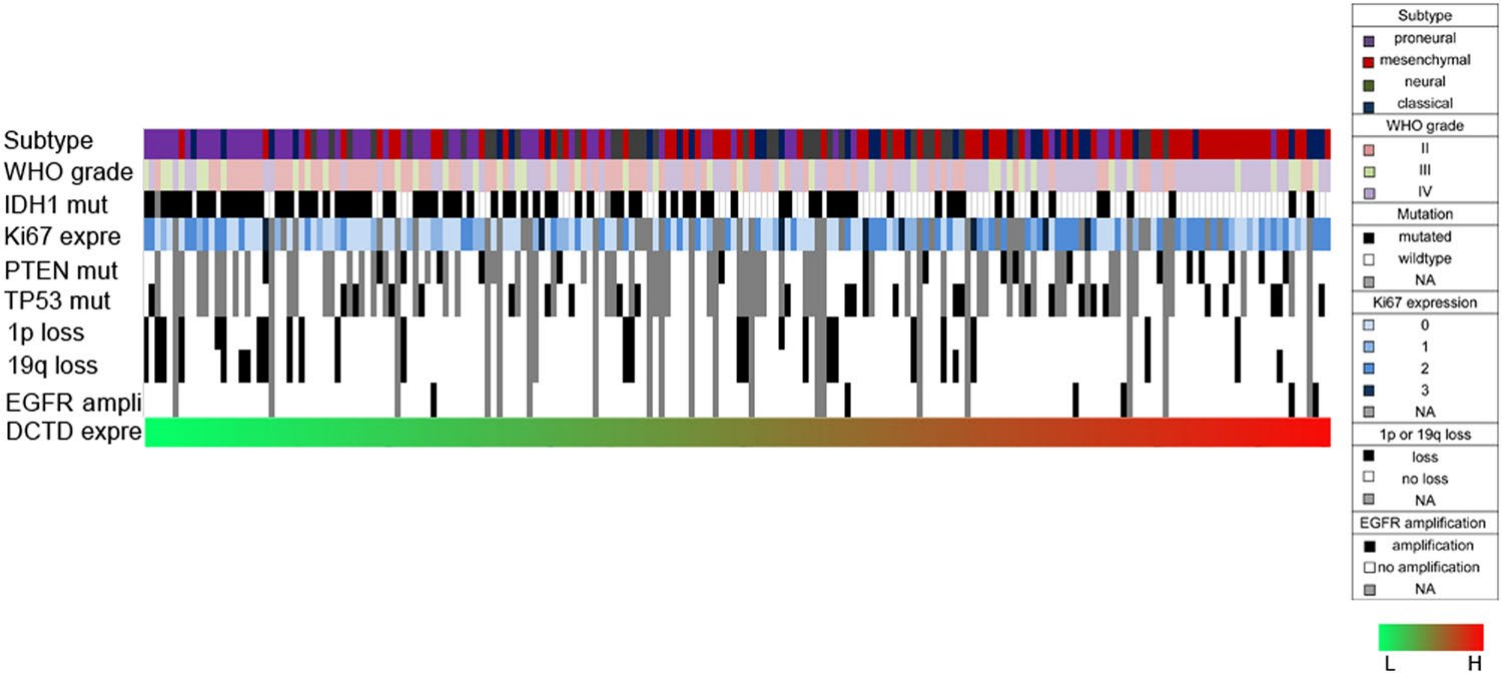
Correlations of *DCTD* expressing-level with the classical genomic or transcriptional alterations in glioma. Abbreviation: mut = mutation; expre = expression level; ampli = amplification; L = low; H = high.

To further depict the oncogenic features of *DCTD*, we obtained an overview of the correlations between *DCTD* expression level and the genomic or transcriptional alterations contributing to the origin or progression of glioma (Fig. 5). According to the above results, *IDH1* mutations occurred more frequently in glioma with lower *DCTD* expression. The well-known indicator of optimistic outcome, co-deletion of 1p19q gathered in glioma with lower *DCTD* expression. The incidences of malignant factors including Ki67 high-expression, *PTEN* mutation, *TP53* mutation and *EGFR* amplification were higher in glioma with higher *DCTD* expression.

**Figure 6:**
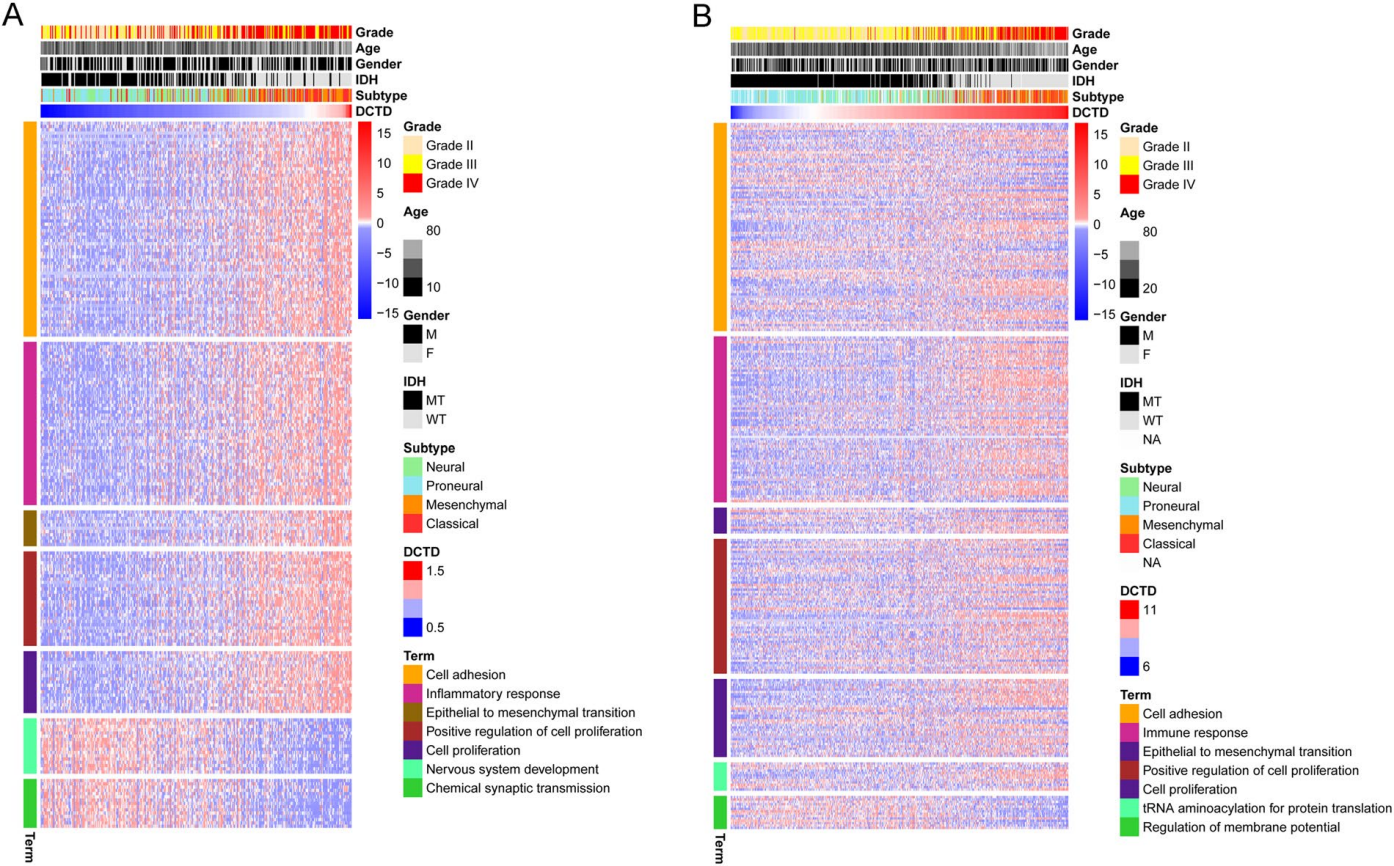
Gene ontology analysis for *DCTD*. Gene ontology analysis for *DCTD* in CGGA (**A**) and TCGA (**B**) RNA sequencing dataset.

To further validate the oncogenic nature of *DCTD*, we annotated the biological differentiation accompanying with alteration of *DCTD* expression level (Fig. 6). The differentially expressing genes between glioma with high and low *DCTD* expression level were separately derived from CGGA or TCGA RNA-sequencing dataset and annotated using the online Database for Annotation, Visualization and Integrated Discovery (DAVID) v6.7. The *DCTD-*related genes were found more frequently involved in the processes of cell adhesion, immune or inflammatory response and epithelial to mesenchymal transition. Considering the critical role of DCTD in genetic material synthesis, we paid extra attention to the proliferation term and found marked enrichment of the *DCTD*-related genes in biological processes of positive regulation of cell proliferation.

## Discussion

We revealed a potentially important gene contributing to glioma origin or malignant progression, and as well a promising prognostic indicator. This is the first report elaborating the pathological and biological role of *DCTD* in glioma. DCTD is a key enzyme in genetic material synthesis, taking charge of conversion of dCMP to dUMP, which is necessary in cancer origin and progression. The critical role of DCTD in genetic material synthesis supports our findings that *DCTD*-related genes were enriched in cell proliferation process.

To depict the role of DCTD in malignant progression of glioma, we performed biological functional annotation of the *DCTD*-related genes. Since the well-known role of DCTD in genetic material synthesis, which is an essential step of cell proliferation and tumor growth, it is unexpected that the *DCTD*-related genes were mostly noted enriched in the processes of cell adhesion and epithelial to mesenchymal transition. To our knowledge, there is no sound evidence indicating that DCTD acts as a stimulator of migration or invasion of tumor cells. The present study indicates a potential role for DCTD in the invasion capacity of glioma cells and our observations warrants further studies.

**Figure 7:**
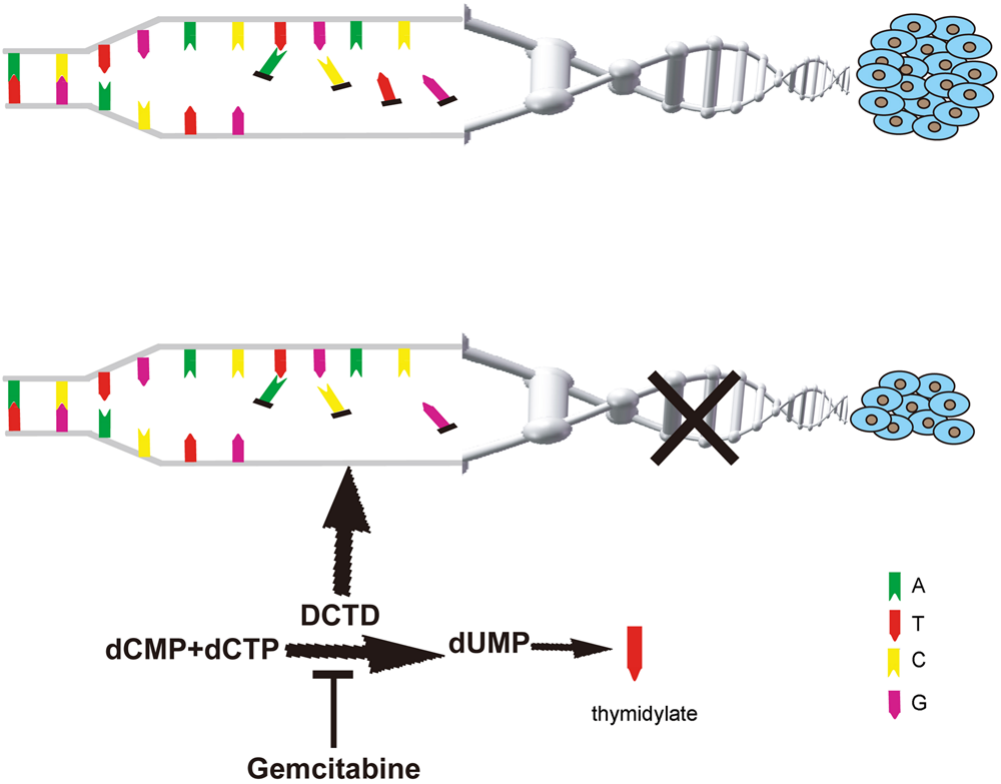
The hypothesis about recommission of gemcitabine as an adjuvant therapy for malignant glioma. The strong proliferation of tumor cells requires active synthesis of genetic material. In the process of genetic material synthesis, the protein product of *DCTD*, dCMP deaminase, plays an important catalyzing role. The ready-made inhibitor of DCTD, gemcitabine, could suppress the synthesis of dTMP and cause a shortage of genetic material, leading to inhibition of the hyperactive proliferation of tumor cells.

Additionally to our proposal that *DCTD* transcriptional level could have an impact on survival rate for patients with malignant glioma, our findings reveal the potential value of DCTD as a therapeutic target as well. The developments of novel therapeutic approaches continue worldwide. The deoxynucleoside analogue gemcitabine had been considered for combination therapy with radiation in GBM^15^. Gemcitabine has been routinely used in the treatment of solid tumors, such as non-small-cell lung cancer (NSCLC), breast and ovarian cancer, bladder cancer and pancreatic cancer^16^. Its active metabolite, gemcitabine triphosphate (dFdCTP) inhibits dCMP deaminase, the protein product of *DCTD* ^17^. Although gemcitabine had been proposed as a promising therapy for GBM in consideration of its effect as a radiosensitizer and favorable feasible properties of permeating the blood–tumor barrier, previous trials focused on the treatment of GBM were stopped after phase 0 evaluation^15^. In the present study, we proposed *DCTD* as a critically important gene in glioma origin and malignant progression. Since gemcitabine is a ready-made inhibitor of *DCTD*, we proposed a hypothesis to support the recommission of gemcitabine as an adjuvant therapy for malignant glioma with high *DCTD* expression (Fig. 7).

## Materials and Methods

### Patients and samples

A total of 302 glioma samples of all WHO grades from CGGA were enrolled in this study. This study was approved by the Institutional Review Boards of Beijing Tiantan Hospital, and written informed consent was obtained from all patients. All methods were performed in accordance with the relevant guidelines and regulations of the Institutional Review Boards. The establishment and management of our CGGA databank have been introduced in our previous publications^18, 19^. Information of Ki67 immunohistochemistry staining^20^, mutations of *IDH1* ^20^, *PTEN* ^21^ and *TP53* ^21^, deletions of 1p and/or 19q^22^ and amplifications of *EGFR* ^19^ are all derived from CGGA database and the detection methods had been described in our previous publications (the refs 19–22).

### Transcriptomic subtype classification

The samples were classified into four transcriptional characteristic subtypes according to the method reported by Brennan *et al*.^23^. The mRNA levels were analyzed according to the Proneural-Neural-Classical-Mesenchymal classes using the signatures published in Verhaak *et al*.^24^, and the single sample Gene Set Enrichment Analysis algorithm (ssGSEA).

### Statistical analysis

Median Absolute Deviation (MAD) was calculated using Matlab. Probes targeting genes that showed the highest variable expression were selected for further analysis. Significance Analysis of Microarray (SAM) was performed using BRB Array Tools developed by Dr. Richard Simon and his team to gain the genes whose transcriptional levels or promoter methylation levels were significantly different between the patients lived for less than 1 year (n = 63) and those lived for more than 3 years (n = 36) after diagnosis using data from the Chinese Glioma Genome Atlas (CGGA). FDR was set as 0.1. Then the gene lists derived based on transcriptional level and promoter methylation level was overlapped. Therefore, we derived a cluster of genes with hypomethylated promoter and a high level of transcription correlated with short survival time. Survival analysis (Cox proportional hazards model) was used to estimate the efficacy of these genes for distinguishing prognosis of all patients with WHO grade III and IV glioma in CGGA (except for the screening group, additional patients whose survival time is between 1 to 3 years were included, and the total number of samples was 178) by BRB Array Tools (Version: 4.3.1, Stable, June 2013). Efficacy of these genes in distinguishing prognosis of GBMs in TCGA dataset was also tested. The genes that could efficiently (nominal significance level of each univariate test was both set as 0.001) indicate OS in CGGA and TCGA datasets were overlapped. Finally, only 7 genes were filtered out.

The diagrams describing the correlations between the expression level of *DCTD* with OS, grades, *IDH1* mutant and subtype were generated using R programming language.

Transcriptome differences between patients with *DCTD* expression higher than median value and those with *DCTD* expression level lower than median value were obtained from CGGA or TCGA databases and gene ontology analysis of the *DCTD* expression level-related genes was performed using online Database for Annotation, Visualization and Integrated Discovery (DAVID) v6.7 (https://david.ncifcrf.gov/).

## Electronic supplementary material

Supplementary Information

## References

[1] Louis DN (2006). Molecular pathology of malignant gliomas. Annu Rev Pathol.

[2] Louis DN (2007). The 2007 WHO classification of tumours of the central nervous system. Acta Neuropathol.

[3] Yang M (2011). L1 stimulation of human glioma cell motility correlates with FAK activation. J Neurooncol.

[4] Jiang T (2016). CGCG clinical practice guidelines for the management of adult diffuse gliomas. Cancer Lett.

[5] Goodenberger ML, Jenkins RB (2012). Genetics of adult glioma. Cancer Genet.

[6] Stupp R, Tonn JC, Brada M, Pentheroudakis G (2010). High-grade malignant glioma: ESMO Clinical Practice Guidelines for diagnosis, treatment and follow-up. Ann Oncol.

[7] Stupp R (2002). Promising survival for patients with newly diagnosed glioblastoma multiforme treated with concomitant radiation plus temozolomide followed by adjuvant temozolomide. J Clin Oncol.

[8] Walker MD (1980). Randomized comparisons of radiotherapy and nitrosoureas for the treatment of malignant glioma after surgery. N Engl J Med.

[9] Li MY (2016). Low c-Met expression levels are prognostic for and predict the benefits of temozolomide chemotherapy in malignant gliomas. Sci Rep.

[10] Weller M (2017). Vaccine-based immunotherapeutic approaches to gliomas and beyond. Nat Rev Neurol.

[11] Gan, H. K. *et al*. Antibody-drug conjugates in glioblastoma therapy: the right drugs to the right cells. *Nat Rev Clin Onco*l. 10.1038/nrclinonc.2017.95. Published online 4 Jul (2017). 10.1038/nrclinonc.2017.95 28675164

[12] Weiner KX (1995). Chromosomal location and structural organization of the human deoxycytidylate deaminase gene. J Biol Chem.

[13] Yan H (2009). IDH1 and IDH2 mutations in gliomas. N Engl J Med.

[14] Noushmehr H (2010). Identification of a CpG island methylator phenotype that defines a distinct subgroup of glioma. Cancer Cell.

[15] Sigmond J (2009). Gemcitabine uptake in glioblastoma multiforme: potential as a radiosensitizer. Ann Oncol.

[16] Toschi L, Finocchiaro G, Bartolini S, Gioia V, Cappuzzo F (2005). Role of gemcitabine in cancer therapy. Future Oncol.

[17] Heinemann V (1992). Cellular elimination of 2′,2′-difluorodeoxycytidine 5′-triphosphate: a mechanism of self-potentiation. Cancer Res.

[18] Hu H (2015). Genome-wide transcriptional analyses of Chinese patients reveal cell migration is attenuated in IDH1-mutant glioblastomas. Cancer Lett.

[19] Liu Y (2014). Multidimensional analysis of gene expression reveals TGFB1I1-induced EMT contributes to malignant progression of astrocytomas. Oncotarget.

[20] Cai J (2014). ATRX mRNA expression combined with IDH1/2 mutational status and Ki-67 expression refines the molecular classification of astrocytic tumors: evidence from the whole transcriptome sequencing of 169 samples samples. Oncotarget.

[21] Liu Y (2016). Integrated analysis identified genes associated with a favorable prognosis in oligodendrogliomas. Genes Chromosomes Cancer.

[22] Yang P (2016). Radiation combined with temozolomide contraindicated for young adults diagnosed with anaplastic glioma. Oncotarget.

[23] Brennan CW (2013). The somatic genomic landscape of glioblastoma. Cell.

[24] Verhaak RG (2010). Integrated genomic analysis identifies clinically relevant subtypes of glioblastoma characterized by abnormalities in PDGFRA, IDH1, EGFR, and NF1. Cancer Cell.

