# Supplementary material for: Gene Expression and Methylation Analyses Suggest DCTD as a Prognostic Factor in Malignant Glioma
Source: Sci Rep. 2017 Sep 14;7:11568. doi: 10.1038/s41598-017-11962-y (PMC5599690; doi:10.1038/s41598-017-11962-y)
Supplement: Supplementary file 1 — Supplementary Information [file 41598_2017_11962_MOESM1_ESM.pdf]

Gene Expression and Methylation Analyses Suggest *DCTD* as a Prognostic Factor in Malignant Glioma

Huimin Hu[a, b, c, d, †], Zheng Wang[a, b, c, d, †], Mingyang Li[a, b, c, d], Fan Zeng[a, b, c, d], Kuanyu Wang[a, b, c, d], Ruoyu Huang[a, b, c, d], Haoyuan Wang[e], Fan Yang[a, b, c, d], Tingyu Liang[a, b, c, d], Hua Huang[a, b, c, d], Tao Jiang[a, b, c, d, *]

Affiliations:

a Department of Molecular Neuropathology, Beijing Neurosurgical Institute, Capital Medical University

b Chinese Glioma Cooperative Group (CGCG)

c Department of Neurosurgery, Beijing Tiantan Hospital, Capital Medical University

d Center of Brain Tumor, Beijing Institute for Brain Disorders

e Southern Medical University

† Contributed equally.

*****Correspondence to: Tao Jiang, Beijing Neurosurgical Institute, Capital Medical University, No.6 Tiantan Xili, Dongcheng District, Beijing 100050, China (E-mail: taojiang1964@163.com).

Supplementary 1

Seven genes were filtered as they were significantly correlated with the survival length of the TCGA GBM patients (the last step of our data analysis pipeline (Figure 1) to search for the OS-correlated critically important genes).

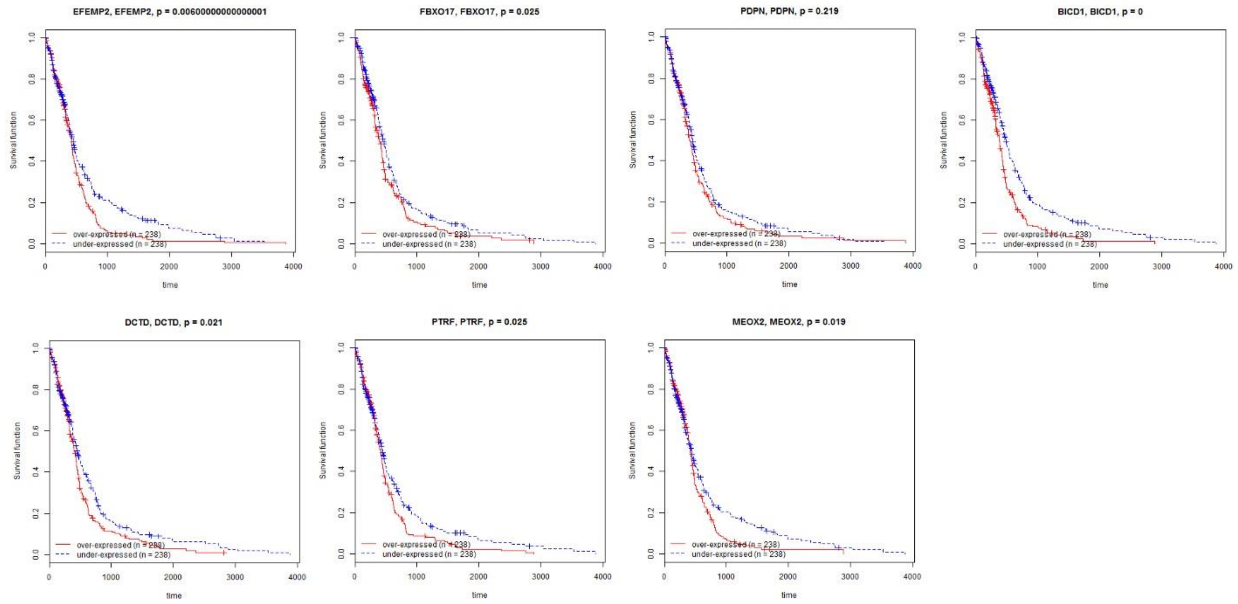
EFEMP2, EFEMP2, p = 0.00600000000000001
FBXO17, FBXO17, p = 0.025
PDPN, PDPN, p = 0.219
BICD1, BICD1, p = 0
DCTD, DCTD, p = 0.021
PTRF, PTRF, p = 0.025
MEOX2, MEOX2, p = 0.019
Survival function
time
over-expressed (n = 238)
under-expressed (n = 238)
